# Exploring Non-Thermal Plasma and UV Radiation as Biofilm Control Strategies against Foodborne Filamentous Fungal Contaminants

**DOI:** 10.3390/foods13071054

**Published:** 2024-03-29

**Authors:** Markéta Kulišová, Michaela Rabochová, Jan Lorinčík, Tomáš Brányik, Jan Hrudka, Vladimír Scholtz, Irena Jarošová Kolouchová

**Affiliations:** 1 Department of Biotechnology, University of Chemistry and Technology, Prague, Technická 5, 166 28 Prague, Czech Republic; irena.jarosova@vscht.cz; 2 Department of Material Analysis, Research Centre Rez, Hlavní 130, 250 68 Husinec-Řež, Czech Republic; michaela.rabochova@cvrez.cz (M.R.); jan.lorincik@cvrez.cz (J.L.); 3 Faculty of Biomedical Engineering, Czech Technical University in Prague, nám. Sítná 3105, 272 01 Kladno, Czech Republic; 4 Research Institute of Brewing and Malting, Lípová 15, 120 44 Prague, Czech Republic; branyik@beerresearch.cz; 5 Department of Physics and Measurements, Prague, University of Chemistry and Technology, Prague, Technická 5, 166 28 Prague, Czech Republic; jan.hrudka@vscht.cz (J.H.); vladimir.scholtz@vscht.cz (V.S.)

**Keywords:** non-thermal plasma, UV radiation, biofilm, filamentous fungi

## Abstract

In recent years, non-thermal plasma (NTP) has emerged as a promising tool for decontamination and disinfection within the food industry. Given the increasing resistance of microbial biofilms to conventional disinfectants and their adverse environmental effects, this method has significant potential for eliminating biofilm formation or mitigating the metabolic activity of grown biofilms. A comparative study was conducted evaluating the efficacy of UV radiation and NTP in eradicating mature biofilms of four common foodborne filamentous fungal contaminants: *Alternaria alternata*, *Aspergillus niger*, *Fusarium culmorum*, and *Fusarium graminearum*. The findings reveal that while UV radiation exhibits variable efficacy depending on the duration of exposure and fungal species, NTP induces substantial morphological alterations in biofilms, disrupting hyphae, and reducing extracellular polymeric substance production, particularly in *A. alternata* and *F. culmorum*. Notably, scanning electron microscopy analysis demonstrates significant disruption of the hyphae in NTP-treated biofilms, indicating its ability to penetrate the biofilm matrix, which is a promising outcome for biofilm eradication strategies. The use of NTP could offer a more environmentally friendly and potentially more effective alternative to traditional disinfection methods.

## 1. Introduction

Earth hosts a vast ecosystem abundant with millions of fungi, engaged in various biological processes. Among them, only approximately 400 species are known to cause human diseases [1,2]. Most microorganisms in the external environment possess the ability to form biofilms, which are associated with approximately 65% of human microbial infections [3]. Biofilms, distinctive formations composed of living cells immersed in an exopolymer, provide physical protection against antifungal agents, disinfectants, and biocidal agents through the formation of an extracellular matrix. The cells within biofilms exhibit resistance to antifungal and antimicrobial agents due to the constitutive up-regulation of efflux pumps and altered metabolic conditions, such as persistent cells inactive metabolically. Furthermore, infections are complicated by robust, inducible gene networks encoding proteins that confer tolerance or resistance to many available antifungal drugs [4,5]. The mechanism of biofilm formation in filamentous fungi differs from that of other microbial biofilms due to its more complex structure, which incorporates fungal hyphae in addition to the extracellular matrix. Hyphae play a crucial role in the formation of the 3D biofilm structure [6,7]. In recent years, there has been an increase in fungal infections, with mycotic diseases responsible for more than 10 million human deaths per year, which presents a significant global health challenge [8,9].

While the emergence of resistant fungal biofilms poses a significant concern, scientific research has focused mainly on the biofilms formed by yeast cells, particularly within the *Candida* genus [10,11,12]. There is a notable scarcity of studies addressing the more intricate biofilms of filamentous fungi, which can present substantial challenges not only in the healthcare sector but also in other industries. Filamentous fungi harbor diverse adaptive mechanisms encoded in their genome, facilitating their pathogenic behavior in the human host [13,14]. The formation of biofilms represents a virulence factor that supports the creation and persistence of infections [15]. Concern about the biofilms formed by filamentous fungi extends beyond the healthcare system to the food industry. Fungi can proliferate on various surfaces, causing not only economic losses but also health issues associated with mycotoxin production [16,17]. Reports indicate an annual average loss of 5 to 10% in global food production due to filamentous fungi [18]. Food contamination can occur at various stages, including the pre-harvest, storage, and production steps [19,20], through direct contact with food products or indirectly, such as through the water used in production [21,22]. Due to their high resistance to sanitation practices, the biofilms formed by filamentous fungi compromise the shelf life and safety of food products [23,24].

Disinfection technologies that are effective against microbes at room temperature include high-power ultrasound [25], ozone [26,27], magnetic field oscillation [28], pulsed electric fields [29], ultraviolet radiation, and high hydrostatic pressure [30]. These processes exhibit efficacy at room temperature, minimizing any adverse impact on food quality. Advanced non-thermal disinfection methods, such as non-thermal plasma (NTP), are still not widely utilized. NTP can serve as a complementary or sole alternative for reducing the microbial load in both food products and packaging materials [31,32]. This technology is also suitable for the healthcare environment, as the combination of various sanitation methods with different mechanisms of action is proven effective in limiting the development of microbial resistance to major antibiotic classes and broadening the spectrum of antimicrobial efficacy [33]. Reactive oxygen and nitrogen particles, generated during the NTP’s action, significantly affect various enzyme activities. Sulphur amino acids undergo oxidation, disulfide bonds break, and aromatic amino acids undergo modification, affecting the primary structure [34,35]. The key mechanisms of biofilm deactivation by NTP include a loss of cell membrane integrity, a reduction in biofilm thickness, and a decrease in cellular metabolic activity. The deep penetration of reactive particles into the biofilm leads to antimicrobial effects due to the formation of secondary reaction products at the biological interface [36,37]. Reactive nitrogen particles destroy proteins, lipids, and nucleic acids within the cell [38]. NTP technology is gaining attention due to its energy efficiency and environmentally friendly and sustainable approach [39].

UV radiation, a widely employed non-contact method for surface decontamination, is currently utilized to suppress biofilms [40]. Its mechanism induces DNA and RNA changes, causing irreversible damage to microorganisms [41,42]. Operating within the 190–280 nm wavelength range, the FDA-approved wavelength for use in food products and juices is 254 nm [43]. The effective eradication of filamentous fungi depends on the duration, dose, the initial concentration of biological agents, and the presence of interference factors such as organic deposits or biofilms in the environment [44,45]. Fixed UV light sources pose technical challenges, including operator safety concerns due to UV exposure, adaptive repair mechanisms in treated fungi, and the shielding effect of spore pigmentation, which absorbs UV light at or near the 254 nm wavelength, protecting fungi [46]. Both UV and NTP offer the advantage of not leaving chemical residues after their application and of not producing heat [47,48]. Simultaneously, these technologies have the potential to deactivate foodborne microorganisms without compromising the nutritional value of food products [40].

This study evaluates the impact of UV radiation with varying exposure times and compares it with the effects of NTP on the mature biofilms of four important foodborne mycotoxigenic fungal contaminants: *Alternaria alternata*, *Aspergillus niger*, *Fusarium culmorum*, and *Fusarium graminearum*. The influence of these irradiations on the metabolic activity of the 48-hour cultured biofilms under different cellular nutrient conditions was investigated, and the effects of irradiation on the morphological changes in the biofilms were monitored using SEM microscopy.

## 2. Materials and Methods

### 2.1. Microscopic Filamentous Fungi

The microscopic filamentous fungal strains utilized in this study were obtained from the Collection of Yeasts and Industrial Microorganisms (DBM) at UCT Prague. Specifically, the strains *Alternaria alternata* DBM 4004, *Aspergillus niger* DBM 4054, *Fusarium culmorum* DBM 4044, and *Fusarium graminearum* DBM 4344 were used. These strains were inoculated on potato dextrose agar (PDA, VWR Chemicals, Atlanta, GA, USA) and cultivated statically at 26 °C for 5 days. Subsequently, Petri dishes containing the cultured strains were stored at 4 °C.

### 2.2. Spore Suspension Preparation

The mature fungal culture grown in the Petri dishes with PDA agar was submerged in a saline solution (0.9% NaCl (*m*/*v*)) to release the spores. Subsequently, a 10 µL aliquot of the spore suspension was placed in a Bürker chamber, and the spore count was assessed using light microscopy. A concentration of 10^5^ spores/mL was used for the subsequent experiments [49]. This concentration was achieved by diluting the spore suspension with a saline solution.

### 2.3. UV Lamp and NTP Generator Specifications

The effect of UV on the selected fungal strains was studied using the Puritec HNS 30 W G13 UV-C low-pressure lamp (Osram, Munich, Germany). Positioned 40 cm above the sample, the lamp emitted UV radiation at an intensity of 7.75 W/m^2^, as specified by the manufacturer.

For the NTP experiments, a generator was developed by the Department of Physics and Measurements at the University of Chemistry and Technology Prague. This generator utilized six direct current bipolar corona discharges arranged in a point-to-ring electrode configuration. Connected to a high-voltage direct current source, the setup induced a negative corona discharge at the point electrode, linked to the negative voltage terminal, and a positive corona discharge at the edge of the ring electrode, connected to the positive voltage terminal. The reactive particles generated by the NTP discharge were transported from the discharge area through the ring electrode and directed to the microscopic fungal strain located within a well of a microtiter plate. The voltage applied to the discharge electrodes was set at U = 6 kV, with each discharge carrying a current of I = 100 µA. Ambient air at atmospheric pressure served as the working gas for electric discharge.

### 2.4. Metabolic Activity Measurement

To evaluate the effectiveness of NTP/UV on the biofilms of filamentous microscopic fungi, the metabolic activity was measured using the MTT (3-[4,5-dimethylthiazol-2-yl]-2,5 diphenyl tetrazolium bromide) spectrophotometric assay following a protocol described by [49]. After the 48 h incubation period, the cell suspension was removed from the microtiter plate wells, and the wells were rinsed twice with phosphate-buffered saline (PBS, pH = 7.4). Next, 600 μL of glucose solution (57 g/L in PBS), 500 μL of MTT solution (Acros Organics, Waltham, MA, USA, 1 g/L in PBS), and 150 μL of menadione solution (Merck Life Science, Rahway, NJ, USA, 0.11 g/L in PBS) were added to each well. The microtiter plate was then incubated on a shaker for 3 hours in the dark (26 °C, 75 rpm). Following incubation, 1 mL of a dissolving solution was added to dissolve the purple formazan crystals generated as a result of the metabolic activity. This solution consisted of 40% dimethylformamide (*v*/*v*) in a solution of 2% acetic acid (*v*/*v*) solution diluted in PBS, along with 16% sodium dodecyl sulphate (*m*/*v*). The plate was further incubated on a shaker for 30 min in the dark (26 °C, 150 rpm). Finally, 100 μL from each well was transferred to a 96-well microtiter plate, and the absorbance was measured at 570 nm.

### 2.5. Monitoring the Effect of NTP and UV Radiation on Fungal Biofilms

**Figure 1:**
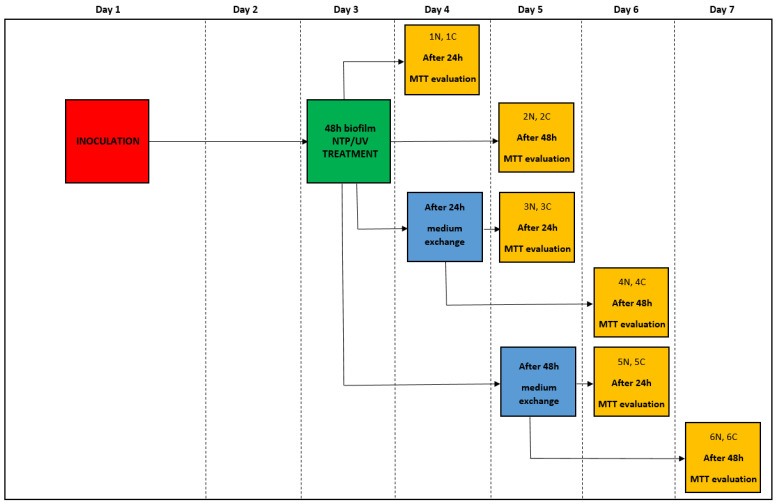
Schematic overview of the experiment with NTP/UV-treated 48 h cultivated biofilm; green—NTP/UV treatment, blue—PDB medium exchange, yellow—metabolic activity evaluation; N—treated sample, C—control sample.

To study the effects of NTP/UV on the biofilms of filamentous microscopic fungi, flat-bottomed 24-well sterile polystyrene microtiter plates (TPP, Trasadingen, Switzerland) were used. Each well of the microtiter plate was filled with 1 mL of a diluted spore suspension containing a concentration of 10^5^ spores/mL and 1 mL of potato dextrose broth (PDB, VWR Chemicals, Radnor, PA, USA, 48 g/L). The plates were then statically cultured for 48 h at 26 °C. Following incubation, the suspension was removed, and the biofilm layer at the bottom of the well was exposed to either NTP treatment (90 min) or UV treatment (60 s, 30 min, 60 min, 90 min). Subsequently, the suspension was gently returned to the wells. To assess the resistance of the fungal biofilms to NTP/UV, six different scenarios were created for each fungal strain (each measured in four replicates). Metabolic activity was quantified using the MTT method, with equivalent control wells (non-NTP/UV-treated) included for each measurement. Throughout the experiment, the original PDB medium was periodically replaced with fresh medium to mitigate the impact of nutrient limitations on the biofilms’ metabolic activity. The entire experimental process, which focused on one strain of filamentous fungi, lasted 5 days. For clarity, a schematic overview of the experiments is provided in Figure 1 for clarity.

### 2.6. Preparation of NTP/UV Treated Biofilms for SEM

The samples for scanning electron microscopy (SEM) were prepared following the procedure outlined in Section 2.5, after which they were treated with NTP/UV for 90 min. Next, a 10 µL aliquot of the suspension was transferred to a silicon plate suitable for SEM microscopy. The samples were allowed to air-dry, and SEM analysis was performed within a 24 h time frame. The control spore samples, which were not subjected to NTP/UV exposure, were also analyzed using SEM.

### 2.7. SEM Microscopy

For this research, the focused ion beam scanning microscopy (FIB-SEM) instrument (LYRA3, TESCAN GROUP, a.s., Brno, Czech Republic) at the Research Centre Rez was employed. This FIB-SEM system combines a field electron emission source with a focused ion beam source.

The dehydrated fungal biofilm samples underwent a coating process with a 5 nm thick platinum film using a Leica EM ACE600 e-beam coater. Platinum was applied at an angle of 45 degrees and at a pressure of 7 × 10^−5^ mbar. Platinum was chosen over gold because of its smaller grain size in the thin film. SEM imaging of the fungal biofilms was performed with an e-beam acceleration voltage of 20 kV and a current of 465 pA, while maintaining a working distance of 9 mm. Secondary electrons were detected using an Everhard–Thornley-type detector. The image acquisition was carried out with a dwell time of 32 μs per pixel and an image resolution of 1024 × 1024 pixels, resulting in a frame time of 33.5 s.

### 2.8. Statistical Analysis

Statistical analysis was conducted using RStudio, employing One-Way Analysis of Variance (ANOVA) with Tukey’s post hoc test and Correlation Analysis to compare and assess the metabolic activity measurements. In instances of non-normal distributions, the data were transformed to achieve normal distributions by applying a natural logarithm before the statistical analysis. Dixon’s Q test was utilized to identify outliers in the data obtained. Four parallel determinations with three independent repetitions were made, ensuring that the deviation between all the measurements was less than 5%.

## 3. Results

### 3.1. The Impact of UV Radiation on the Metabolic Activity of Microscopic Filamentous Fungal Biofilms

**Figure 2:**
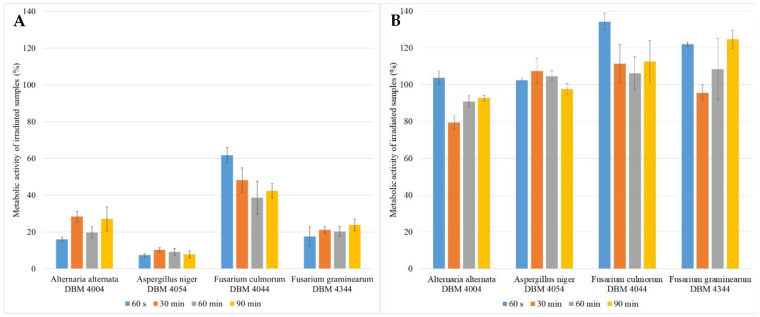
Comparison of the metabolic activity of 48 h grown fungal biofilms treated with UV radiation for 60 s (UV dose = 465 J/m^2^), 30 min (UV dose = 13,950 J/m^2^), 60 min (UV dose = 27,900 J/m^2^), 90 min (UV dose = 41,850 J/m^2^); (**A**)—evaluation after 24 h UV treatment, (**B**)—evaluation after 48 h UV treatment.

The study investigated the impact of various UV doses (60 s, 30 min, 60 min, and 90 min) on each microorganism in multiple experimental setups. The biofilms were cultivated for 48 h, then exposed to specific radiation doses, and subsequently cultured for an additional 24 or 48 h to simulate the effects of UV radiation in medical and food processing settings, while considering the generation time of filamentous fungi (Figure 2). Control experiments were conducted for all the setups. Figure 2A (24 h after UV exposure) indicates that UV radiation had the least effect on the *F. culmorum* biofilm, resulting in a maximum 60% decrease in the metabolic activity of its cells. In contrast, UV radiation was the most effective in the biofilm of *A. niger*, with its metabolic activity dropping to only about 10% after treatment. However, Figure 2B shows that after an additional 24 h (48 h after UV exposure), the metabolic activity of the cells returned to or exceeded the same level as that of the control cells. This suggests that, given enough time, the metabolic activity of biofilm cells can recover to its original level through reparative processes. This recovery appears to be independent of the duration of irradiation, that is, the UV dose, except for with *F. culmorum*, where the biofilm cells showed a decrease in metabolic activity with an increasing radiation dose compared to the controls.

**Figure 3:**
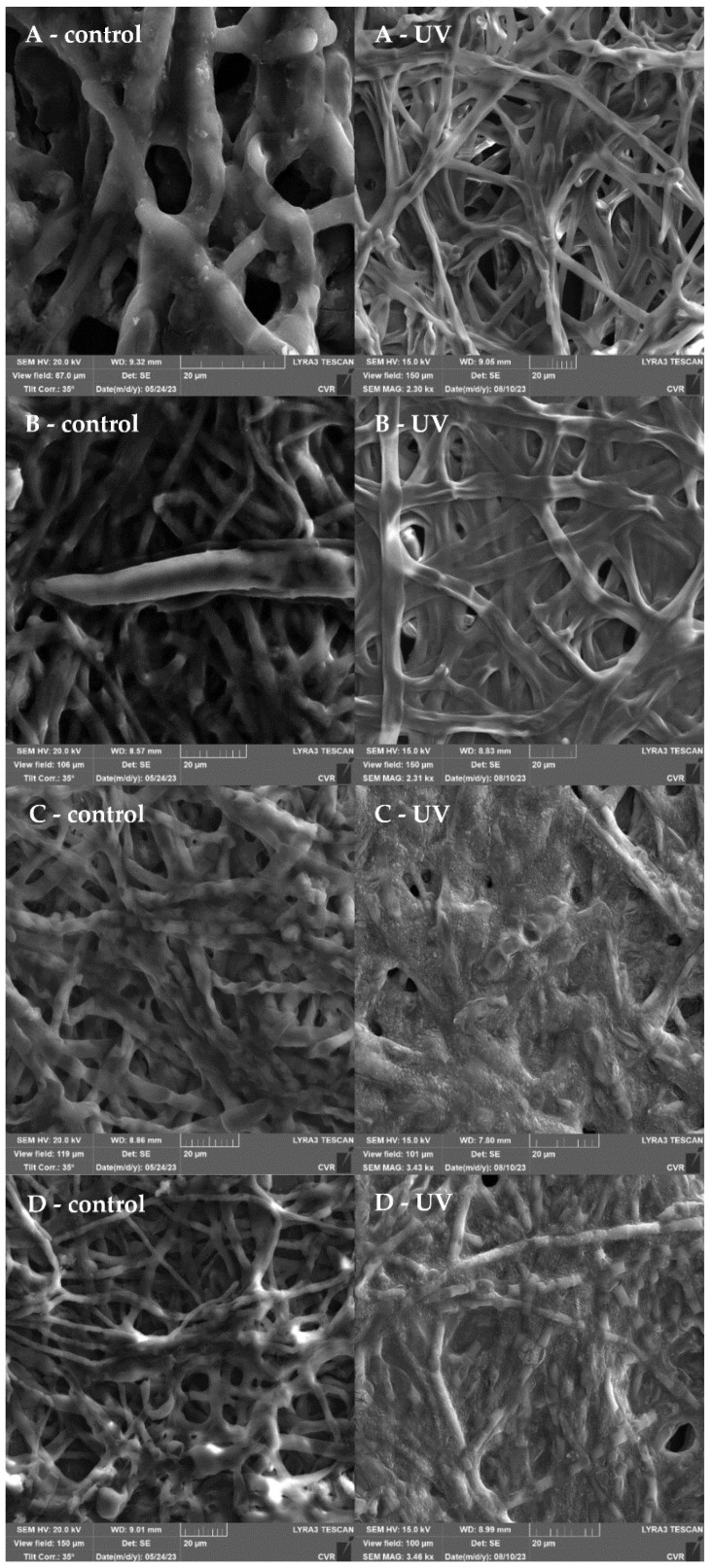
Effect of UV radiation (90 min) on 48 h grown biofilm; (**A**)—*A. alternata* DBM 4004; (**B**)—*A. niger* DBM 4054; (**C**)—*F. culmorum* DBM 4044; (**D**)—*F. graminearum* DBM 4344.

The impact of the highest UV dose (90 min treatment) was further examined using SEM, as illustrated in Figure 3. The response of the filamentous fungi biofilms to UV irradiation is characterized by a significant development of extracellular polymeric substances (EPS). An increase in the size of the biofilm cells is observed in *A. niger* (Figure 3B) and *F. graminearum* (Figure 3D). In *A. alternata* (Figure 3A), a denser network of UV-treated biofilm cells is evident compared to the controls. In both *Fusarium* species (Figure 3C,D), the production of EPS after UV irradiation is so substantial that the hyphae are nearly completely engulfed by this structure.

### 3.2. Comparison of the Effect of UV Radiation and NTP on Microscopic Filamentous Fungal Biofilms

Given the ineffectiveness of UV irradiation even after 90 min of exposure, the same duration was used to evaluate the impact of NTP on fungal biofilms. The experiments were extended to scenarios pertinent to the food industry, where inadequate removal of microbial contamination allows microbial cells to access new nutrient sources. The results cover six experimental variations (Figure 1), in which a 48 h biofilm underwent UV or NTP treatment for 90 min. Subsequently, the metabolic activity of the biofilm cells was evaluated 24 or 48 h after exposure and presented as variants 1 and 2, respectively. An additional experiment involved the introduction of a new source of nutrients. In this scenario, the biofilm was irradiated, and the medium was replaced with fresh medium after 24 h, followed by measuring the metabolic activity of the cells after an additional 24 or 48 h, called variants 3 and 4, respectively. The final series of experiments involved irradiating the biofilm, changing the medium after 48 h, and measuring the metabolic activity of the biofilm cells after an additional 24 or 48 h, called variants 5 and 6, respectively.

**Figure 4:**
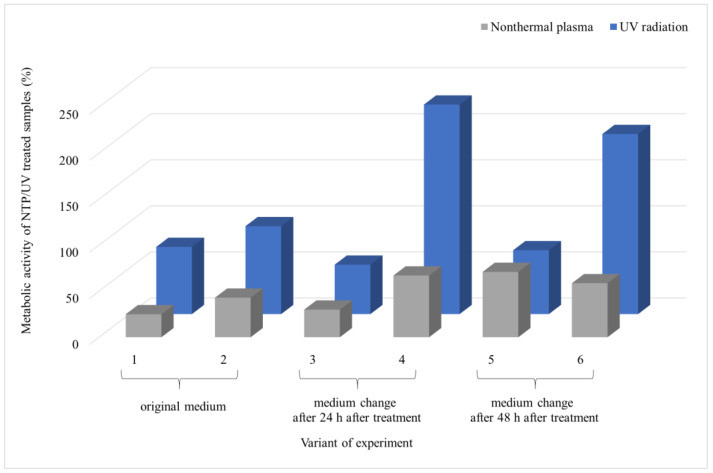
Comparison of the effects of UV radiation and NTP (90-min treatment) on the 48 h grown biofilms of filamentous fungus *A. alternata* DBM 4004. Evaluation time points are indicated for each experimental variant: first and second variants were assessed at 24 and 48 h post-treatment with the original PDB medium; for the third and fourth variants, medium exchange occurred at 24 h post-treatment, followed by evaluations at 24 and 48 h after exchange; for the fifth and sixth variants, medium exchange occurred at 48 h post-treatment, followed by evaluations at 24 and 48 h after exchange. Four parallel determinations with three independent repetitions were made; the deviation of all measurements was less than 5%.

From the results of *A. alternata* (Figure 4), it is evident that NTP was more effective in inhibiting the biofilm growth than UV irradiation. Furthermore, it can be observed that the metabolic activity of the biofilm cells increased over time after exposure to UV and NTP (variants 1 and 2), reaching up to 95% for UV and 43% for NTP compared to the control. Introducing new nutrients 24 h after exposure led to an increase in metabolic activity 48 h after the addition of the nutrients (variant 4), reaching up to 230% relative to the UV control and only 67% of the metabolic activity relative to the NTP control. The introduction of nutrients 48 h after exposure did not significantly impact the trend of increased metabolic activity for UV, but it did affect the metabolic activity of the cells exposed to NTP (variants 5 and 6). There was no elevation in the metabolic activity with an increase in the biofilm age compared to the control; instead, there was a decrease from 71% to 60% between variants 5 and 6, respectively (Figure 4).

**Figure 5:**
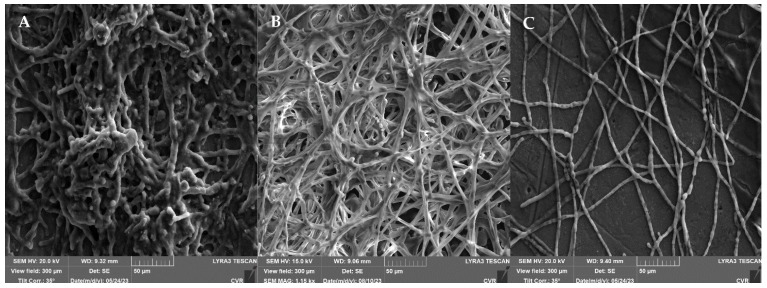
*A. alternata* DBM 4004 biofilm ((**A**)—control, (**B**)—after UV radiation, (**C**)—after NTP).

SEM visualization of the *A. alternata* biofilm (Figure 5) reveals a significant difference in the biofilm structure after exposure to UV or NTP for 90 min (assessed after 24 h post-irradiation). The metabolic activity of the cells after UV irradiation was three times higher compared to exposure to NTP (73% and 25%, respectively), as shown in Figure 4. This observation aligns with the findings obtained using SEM. In the case of NTP, a sparse network of hyphae is visible, with no distinct formation of EPS, and the overall mass of the biofilm is nearly negligible.

**Figure 6:**
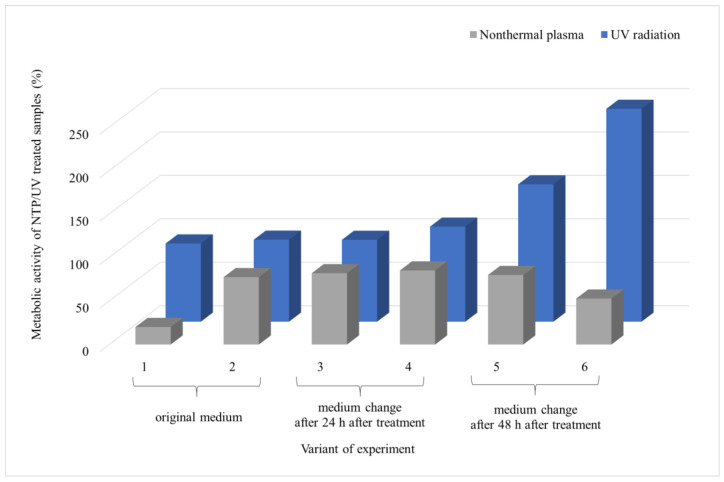
Comparison of the effects of UV radiation and NTP (90 min treatment) on the 48 h grown biofilms of filamentous fungus *A. niger* DBM 4004. Evaluation time points are indicated for each experimental variant: first and second variants were assessed at 24 and 48 h post-treatment with the original PDB medium; for the third and fourth variants, medium exchange occurred at 24 h post-treatment, followed by evaluations at 24 and 48 h after exchange; for the fifth and sixth variants, medium exchange occurred at 48 h post-treatment, followed by evaluations at 24 and 48 h after exchange. Four parallel determinations with three independent repetitions were made; the deviation of all measurements was less than 5%.

The most notable contrast between the effects of UV and NTP on the microorganism *A. niger* is evident in variant 1, as depicted in Figure 6. Here, the impact of NTP is significant, resulting in a reduction in metabolic activity to 20% of that of the control, while with UV, the metabolic activity remained at 80%. The introduction of new nutrients into the biofilm cells after UV irradiation did not affect the decrease in metabolic activity; instead, as the biofilm aged, the metabolic activity of the cells increased, reaching up to 245% compared to the control in variant 6. On the contrary, the biofilm exposed to NTP did not show a significant change in metabolic activity even with the addition of new nutrients, experiencing a decrease of approximately 20% in variant 6 compared to the control. Similarly to the case of *A. alternata*, there was a reduction in the metabolic activity of the cells with the increasing age of the biofilm, regardless of the supply of nutrients (variant 6).

**Figure 7:**
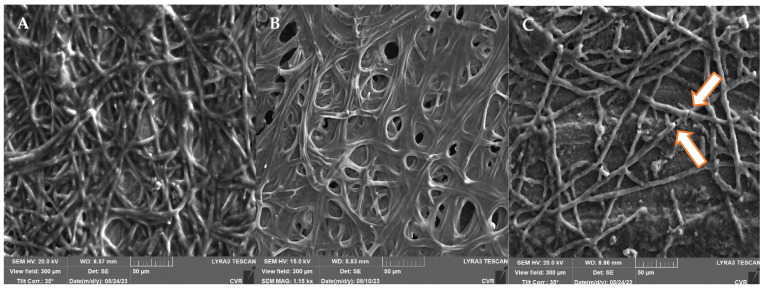
*A. niger* DBM 4054 biofilm ((**A**)—control, (**B**)—after UV radiation, (**C**)—after NTP). Arrow indicated mechanical disruption of the hyphae.

A comparison of the microscopic images (Figure 7) between the *A. niger* control biofilm and UV-treated and NTP-treated biofilms clearly demonstrates the remarkable effectiveness of NTP in reducing the hyphal biomass and minimizing EPS. Mechanical disruption of the hyphae is also evident, with noticeable interruptions in the continuity of the hyphal filaments (Figure 7C, indicated by the arrow). On the contrary, UV exposure resulted in the substantial formation of EPS (Figure 7B).

**Figure 8:**
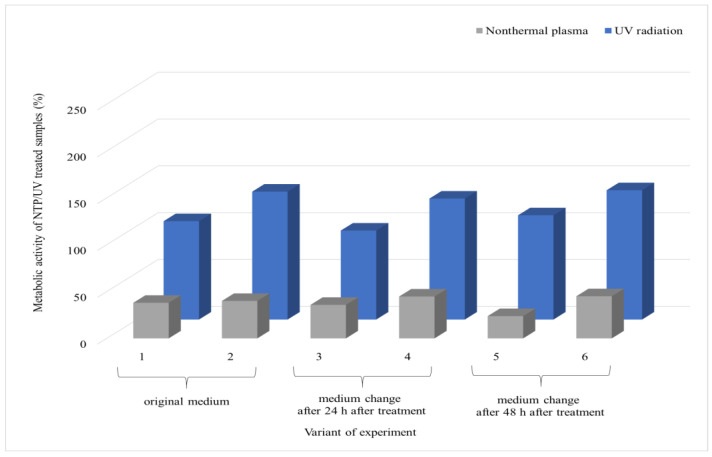
Comparison of the effects of UV radiation and NTP (90 min treatment) on the 48 h grown biofilms of filamentous fungus *F. culmorum* DBM 4004. Evaluation time points are indicated for each experimental variant: first and second variants were assessed at 24 and 48 h post-treatment with the original PDB medium; for the third and fourth variants, medium exchange occurred at 24 h post-treatment, followed by evaluations at 24 and 48 h after exchange; for the fifth and sixth variants, medium exchange occurred at 48 h post-treatment, followed by evaluations at 24 and 48 h after exchange. Four parallel determinations with three independent repetitions were made; the deviation of all measurements was less than 5%.

**Figure 9:**
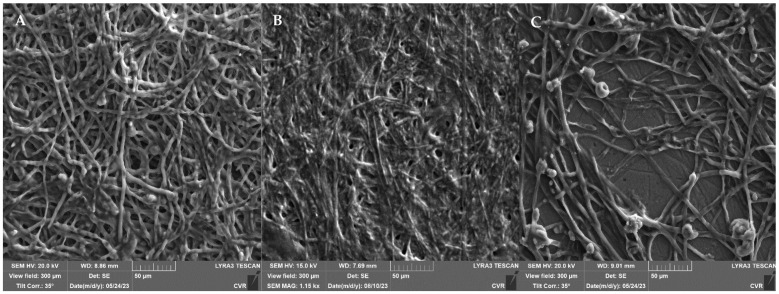
*F. culmorum* DBM 4044 biofilm ((**A**)—control, (**B**)—after UV radiation, (**C**)—after NTP).

UV treatment of the filamentous biofilm of *F. culmorum* showed a minimal impact on the metabolic activity of the biofilm (Figure 8). The SEM images (Figure 9) again depict a significant production of EPS after the UV treatment. On the contrary, NTP substantially reduced the metabolic activity of the biofilm cells across all the experimental treatments, ranging from 23% to 45% compared to the control. Additionally, visualization of the biofilms after NTP treatment indicates a notable inhibition of biofilm formation without the presence of EPS.

**Figure 10:**
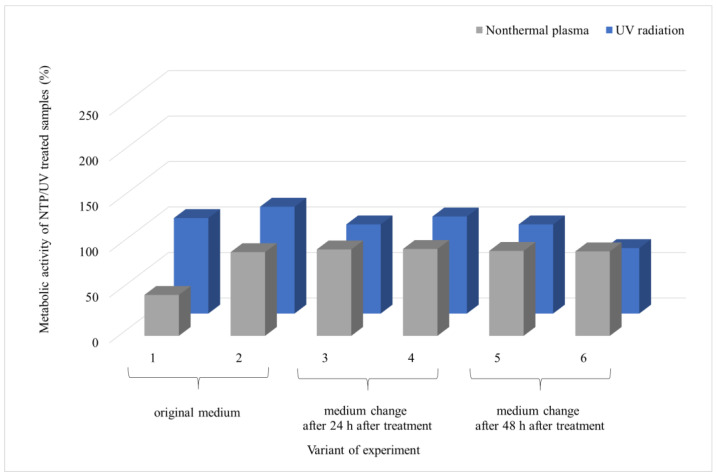
Comparison of the effects of UV radiation and NTP (90 min treatment) on the 48 h grown biofilms of filamentous fungus *F. graminearum* DBM 4004. Evaluation time points are indicated for each experimental variant: first and second variants were assessed at 24 and 48 h post-treatment with the original PDB medium; for the third and fourth variants, medium exchange occurred at 24 h post-treatment, followed by evaluations at 24 and 48 h after exchange; for the fifth and sixth variants, medium exchange occurred at 48 h post-treatment, followed by evaluations at 24 and 48 h after exchange. Four parallel determinations with three independent repetitions were made; the deviation of all measurements was less than 5%.

Both UV and NTP were the least effective in the biofilm of *F. graminearum*, as illustrated in Figure 10. The metabolic activity of the biofilm cells after the UV treatment closely mirrored the control values, with the only exception in variant 6. In this case, involving the supply of fresh nutrients after 48 h after irradiation, followed by another 48 h of cultivation, the metabolic activity decreased to 72% compared to the control. NTP demonstrated its highest impact in terms of a reduction in metabolic activity in variant 1 (the sample evaluation after 24 h after irradiation), with a value reaching 45% compared to the control. In all the other types of experiments, the metabolic activity of the cells did not decrease below 90% compared to the control.

**Figure 11:**
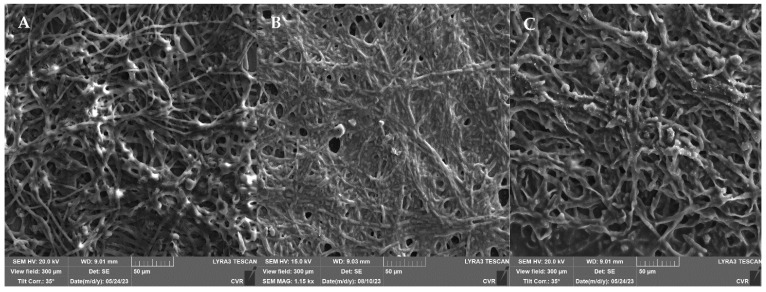
*F. graminearum* DBM 4344 biofilm ((**A**)—control, (**B**)—after UV radiation, (**C**)—after NTP).

The SEM imaging of the *F. graminearum* biofilm (Figure 11) after UV treatment reveals significant EPS development. On the other hand, the image after NTP treatment closely resembles the control sample.

In summary, NTP showed greater effectiveness on the mature biofilms of the filamentous fungi compared to UV irradiation. The only exception was *F. graminearum*, which showed a modest decrease in biofilm metabolic activity, up to 10% compared to the control, which was associated with the SEM images, where the control sample and the NTP-treated sample behaved similarly.

## 4. Discussion

### 4.1. Impact of UV Radiation on the Metabolic Activity of Microscopic Filamentous Fungal Biofilms

The contamination of various synthetic surfaces plays a critical role not only in healthcare settings, where it facilitates the cross-transmission of pathogens, leading to subsequent patient colonization [41], but also in the food industry, where it poses a threat to food safety due to contact with contaminated surfaces during the production process [50,51]. The ability to survive in adverse conditions is not unique to bacteria but extends to filamentous fungi as well [52,53,54]. Today, light therapy, particularly UV radiation, represents a commonly employed approach to eradicating these microorganisms, both in medicine, for the treatment of surface infections [55], and within the food industry [43]. The effects of UV radiation are well documented, causing structural changes in DNA and RNA, disrupting transcription and replication processes, and ultimately leading to cell death [56]. However, UV radiation has adverse effects on certain materials, such as plastics [57], limiting its applicability in medicine. Despite intensive research efforts, the challenge of eliminating microorganisms from various surfaces persists. Emerging techniques, such as the utilization of non-UV visible light with a wavelength of 405 nm, diminish the presence of pathogenic bacteria, yeasts, and molds, although they fall short of achieving complete eradication. Consequently, these microorganisms are progressively developing resistance to conventional disinfection and eradication methods [55].

The paucity of studies examining the efficacy of UV radiation in eliminating filamentous fungi is notable [50]. Byun et al. applied UV radiation for up to 2 h but did not achieve complete effectiveness against *A. flavus* and *A. parasiticus*. Moreover, their focus was solely on the spores, with a mere 1 h interval between the inoculation and UV treatment [58]. Our findings clearly illustrate that the eradication of fungal biofilms depends not only on the duration of the exposure to UV radiation but also on the evaluation method, including the time interval between irradiation and the evaluation of metabolic activity (Figure 2A,B), along with the specific type of filamentous fungi. Although numerous studies have evaluated the lethal impact of UV radiation immediately after application, it is recognized that cells require time to utilize repair mechanisms subsequent to exposure to irradiation-induced radicals [59,60]. Another contributing factor to increased resistance in filamentous fungi is the formation of microconidia and particularly arthroconidia, which exhibit greater resilience to stress conditions and require prolonged dormancy before transitioning to an active hyphal state [61]. This phenomenon is evident in our results, where a substantial contrast in the metabolic activity of the biofilm cells evaluated after 24 (Figure 2A) and 48 h (Figure 2B) after irradiation is apparent. The studied filamentous fungi belong to the priority pathogenic species characterized by a high resistance to antifungals [62] due to the robust capacity for molecular and cellular repair mechanisms [63]. Species capable of surviving on seemingly dry surfaces [51], such as filamentous fungi, demonstrate greater success in developing tolerance to diverse stressors.

The impact of UV radiation on mature biofilms, as described in [64], was investigated using SEM (Figure 3). Our findings unequivocally reveal that exposure to UV radiation significantly induced the production of an extracellular matrix (ECM) compared to the control group. In particular, for *A. alternata* and *A. niger*, we observed the formation of condensed ECMs and film-like ECMs with deeply embedded hyphae, consistent with the descriptions in Rayon-Lopez’s research [65]. Additionally, Rayon-Lopez describes the occurrence of vesicular ECMs in *A. terreus*, a phenomenon which we observed in the *F. culmorum* and *F. graminearum* fungi post-UV radiation, accompanied by abundant mycelia embedded into the ECM. Comparative analysis of the metabolic activity, determined using MTT with SEM results, revealed a robust development of ECMs, which serve a protective function, despite a decline in metabolic activity after 24 h of exposure. Therefore, a subsequent increase in metabolic activity after 48 h appears logical. The literature suggests that one potential mechanism of tolerance to UV radiation could be cell enlargement [59,66]. This phenomenon was observed in *A. niger* and *F. graminearum*, with a significant cell enlargement ranging from 20% to 35% compared to the control group. On the contrary, no discernible increase in cell size was observed in *A. alternata*, while in *F. culmorum*, assessment of alterations in size was precluded due to the cells embedding into a dense ECM matrix.

With the limited effectiveness of UV radiation, there is a growing interest in exploring novel approaches, including the combination of UV radiation with other antimicrobial agents or the utilization of a combination of radiation with biocontrol agents [67]. Furthermore, special emphasis is placed on environmentally friendly methods that aim to minimize waste production and mitigate negative environmental impacts [68,69]. Consequently, in this study, we focused on comparing the effects of two environmentally friendly methods: UV radiation and NTP. Both these light-based techniques are considered low-risk methods for the development of resistance or tolerance, as they effectively target the destruction of various cellular structures, including cell membranes, proteins, lipids, and DNA [59].

### 4.2. Comparison of the Effect of UV Radiation and NTP on Microscopic Filamentous Fungal Biofilms

Currently, UV radiation is still most often used to sterilize work surfaces, container casings, and rooms in food plants. UV radiation is relatively highly effective against bacteria and yeast spores and biofilms, which has been discussed in several studies [70,71]. However, with some microorganisms, biofilm formation will only be suppressed by UV radiation, not completely stopped. Since microscopic fungi are more complex and resistant structurally and functionally than bacteria and yeast, the effect of UV radiation may not be successful enough to inactivate them. Due to these facts, alternatives to biofilm eradication are being sought. One possibility is the use of low-temperature plasma. The study by Soušková et al. points out that yeast and filamentous fungi are more resistant to NTP’s action than bacteria [70]. NTP is characterized by a varying effectiveness in inactivating filamentous fungi, depending on the target fungal species, the types of gases used to create the plasma, the distance between the plasma device and the sample, and the treatment time. The same conclusion was reached by Sakudo et al., who found that after 5 min of NTP treatment using a nitrogen plasma device, the viable cell count of *Aspergillus brasiliensis* was not significantly affected, while *Salmonella enterica* was completely inactivated. Triple the time was required to reduce the viability of *Aspergillus brasiliensis* cells. Thus, compared to bacteria, an extended treatment time must be used to inactivate filamentous fungi [72,73]. In these studies, they used NTP to reduce the viability of only individual cells from the given strains, not biofilms. Because of this, in our experiment, a longer period of NTP action was chosen, specifically 90 min, for the action of NTP on the filamentous fungal biofilms since they are, by their very nature, much more resistant than individual cells.

Soušková et al., reported that the sensitivity of microscopic filamentous fungi to NTP varies between the observed species (*Aspergillus oryzae*, *Cladosporium sphaerospermum*, and *Penicillium crustosum*), while no significant differences in sensitivity to NTP were found among the observed bacterial strains. The genus *Aspergillus* showed the greatest resistance to NTP inactivation [70]. This phenomenon was also observed in our experiments with *A. niger*, in which increased resistance to NTP was also observed compared to the species *A. alternata* and *F. culmorum*.

Our study also found that NTP was more effective against *F. culmorum* than against *A. alternata*, consistent with the findings of Zahoranova et al. In their study, they studied the efficiency of NTP on *A. flavus*, *A. alternati*, and *F. culmorum* derived from maize plants, and *F. culmorum* was found to be the most sensitive to NTP treatment [74]. Hoppanová et al. studied the impact of NTP on the mycelia of *A. parasiticus*, where disruption of the integrity of the hyphal mycelia was reported after NTP treatment. As observed in Figure 7, similar significant changes in the morphology and integrity of *A. niger*’s hyphae were visible in this study after NTP treatment, influencing the amount of EPS and leading to significant dehydration, which is connected with the findings mentioned by Hoppanová et al. [75,76].

When comparing the effects of UV radiation and NTP using SEM analysis, there is a substantial difference in the impact of both types of radiation (Figure 5, Figure 7, Figure 9 and Figure 11). The mycelial hyphae of the control samples are similar to those after UV irradiation, displaying smooth, well-developed hyphae with an even surface, devoid of breaks or visible morphological changes. In *A. niger*, after UV irradiation (Figure 7B), a significant development of EPS is evident as a response to the stress effect on cells. In contrast, the hyphae after exposure to NTP exhibited strong dehydration on their surfaces, numerous pits, breaks, and a noticeably weaker and significantly reduced layer of EPS, observed in both *A. alternata* and *A. niger*. A similar disruption of the hyphal structures after NTP treatment has been documented [77]. NTP could induce these morphological alterations by generating reactive particles, predominantly O_3_ in the specific generator used in the presented study, initiating a cascade of subsequent cellular damage.

It is crucial to note that although the results of NTP’s efficiency against foodborne fungal contaminants show promise, the application of these findings in practical contexts requires thorough testing to verify its safety, efficacy, and economic feasibility.

## 5. Conclusions

In summary, our study addresses the resilience of filamentous fungal biofilms and evaluates the effectiveness of UV radiation and NTP in their eradication. Our findings reveal that while UV radiation exhibits varying efficacy depending on the exposure duration and fungal species, NTP induces significant morphological alterations in biofilms, disrupting hyphal structures, and reducing extracellular polymeric substance production. These results emphasize the need for further research into filamentous fungal biofilms and the formulation of effective eradication strategies, particularly in industries such as food production, where exposure is high. Additionally, harnessing the potential of NTP holds promise for combating the resilience of these biofilms while minimizing the environmental impact. In conclusion, our findings contribute to the growing body of knowledge surrounding biofilm eradication strategies, facilitating the development of more efficient and sustainable methods to address filamentous fungal biofilm challenges in various sectors.

## Data Availability

The original contributions presented in the study are included in the article, further inquiries can be directed to the corresponding author.
